# Clinical benefit in recurrent glioblastoma from adjuvant NovoTTF-100A and TCCC after temozolomide and bevacizumab failure: a preliminary observation

**DOI:** 10.1002/cam4.421

**Published:** 2015-01-26

**Authors:** Eric T Wong, Edwin Lok, Kenneth D Swanson

**Affiliations:** Brain Tumor Center and Neuro-Oncology Unit, Beth Israel Deaconess Medical Center, Harvard Medical SchoolBoston, Massachusetts

**Keywords:** Bevacizumab, chemotherapy, glioblastoma, NovoTTF-100A

## Abstract

The NovoTTF-100A is a device that emits alternating electric fields and it is approved for the treatment of recurrent glioblastoma. It works by perturbing tumor cells during mitosis as they enter anaphase leading to aneuploidy, asymmetric chromosome segregation and cell death with evidence of increased immunogenicity. Clinical trial data have shown equivalent efficacy when compared to salvage chemotherapies in recurrent disease. Responders were found to have had a lower dexamethasone usage and a higher rate of prior low-grade histology. We treated a series of patients with NovoTTF-100A and bevacizumab alone (*n* = 34) or in combination with a regimen consisting of 6-thioguanine, lomustine, capecitabine, and celecoxib (TCCC) (*n* = 3). Compared to the former cohort, the latter cohort exhibited a trend for prolonged overall survival, median 4.1 (0.3–22.7) months versus 10.3 (7.7–13.6) months respectively (*P* = 0.0951), with one experiencing an objective response with a 50% reduction in tumor size on magnetic resonance imaging despite possessing a larger tumor size at baseline and more severe neurologic dysfunction than the median for either group. These observations illustrate the possibility of improving survival and achieving a response in patients with end-stage recurrent glioblastoma by biasing the tumor toward anti-tumor immunologic response with a combination of NovoTTF-100A and TCCC, as well as the continuation of bevacizumab in order to limit dexamethasone use due to its global immunosuppressive effect on the patient.

## Introduction

Recurrent glioblastoma is a difficult-to-treat tumor due to the high degree of molecular aberrations present in tumor cells as well as the existence of both inherent and iatrogenic immunosuppression 1–3. The standard of care for recurrent glioblastoma is bevacizumab 4,5. The NovoTTF-100A device is another treatment for recurrent glioblastoma approved by the United States Food and Drug Administration (FDA) and it works by emitting alternating electric fields via two pairs of orthogonally positioned transducer arrays placed on the scalp. In the pivotal phase III clinical trial, the NovoTTF-100A device has been demonstrated to have equivalent efficacy when compared to Best Physicians Choice chemotherapy while sparing patients the toxicities associated with systemic chemotherapy 6,7. The electric fields affect cells during mitosis due to disruption of the cytokinetic furrow, which forces aberrant mitotic exit. This results in tetraploidy and aneusomy due to asymmetric chromosome segregation 8,9. These post-mitotic cells subsequently exhibit signs of stress that includes HMGB1 secretion and elevated cell surface expression of calreticulin, which likely make them more readily detected by activated phagocytic immune cells and cleared by the patient's anti-tumor immune response 9. Dexamethasone can induce global immunosuppression, making patients prone to infections and likely disabling their anti-cancer immunity 3. A retrospective analysis of responders in the phase III clinical trial revealed that all responders in the NovoTTF-100A arm had a low dexamethasone burden that statistically varied from the population as a whole, while the chemotherapy cohort did not 10. This further suggested a role for an anti-tumor immune response to affect NovoTTF-100A treatment efficacy.

Bevacizumab, a humanized monoclonal antibody against vascular endothelial growth factor (VEGF), can partially replace dexamethasone for controlling cerebral edema in glioblastoma but it can also counteract VEGF-mediated induction and maintenance of tolerogenic dendritic and regulatory T cells 11,12. The NovoTTF-100A has also been used in conjunction with bevacizumab without overlapping toxicities 13. Here, we report the clinical outcome of patients with recurrent glioblastoma who failed salvage treatments and received a regimen consisting of NovoTTF-100A and bevacizumab with or without TCCC chemotherapy (6-thioguanine, lomustine [CCNU], capecitabine, and celecoxib) 14. We observed a favorable outcome in the former patients compared to the cohort receiving NovoTTF-100A and bevacizumab alone, with a trend for improved overall survival (OS) and an objective response with tumor shrinkage and decreased tumor blood flow.

## Methods

A retrospective chart review was conducted, under an institutional review board-approved protocol at Beth Israel Deaconess Medical Center, on patients treated with NovoTTF-100A and bevacizumab between November 2011 and December 2013. Clinical information, laboratory parameters and neuroradiology data from magnetic resonance imaging (MRI), specifically T1-weighted post-gadolinium (T1Gad), fluid attenuated inversion recovery (FLAIR), diffusion and perfusion images, were extracted and tabulated. The patients were segregated into two cohorts: (i) those treated with NovoTTF-100A and bevacizumab only and (ii) those received NovoTTF-100A, bevacizumab and TCCC. Response to treatment was measured according to the Response Assessment in Neuro-Oncology criteria 15. Progression-free survival (PFS) and OS was measured from the time of application of these treatments to death or last follow up.

NovoTTF-100A treatment performed as described previously 6 and patients were subsequently evaluated in the neuro-oncology clinic on a monthly basis for assessment of neurological status, inspection of scalp for breakdown or reaction, review of compliance data downloaded from the generator, and surveillance head MRI performed periodically every 2 months. Bevacizumab was administered at a dose of 10 mg/kg every 2 weeks. The cohort that received only NovoTTF-100A and bevacizumab underwent MRI assessment every 8 weeks or at the time of neurologic deterioration. The other cohort received TCCC oral multidrug therapy with slight modifications after the publication of its activity by Walbert et al. in 2011 14. TCCC consists of administration of 6-thioguanine at a dose of 80 mg/m^2^ every 6 hour from days 1 to 3, followed by lomustine at a dose of 100 mg/m^2^ orally on day 4. This was followed by capecitabine 825 mg/m^2^ every 12 hour and celecoxib 400 mg every 12 hour from days 11 to 24. The cycle is repeated every 42 days or 6 weeks. Complete blood counts with differential, T lymphocytes subsets, electrolytes, liver function tests and anticonvulsant level were obtained at baseline and repeated after every cycle of treatment. Within each cycle, weekly CBC was obtained to monitor white blood cell and platelet counts. A gadolinium-enhanced head MRI, with concomitant perfusion, diffusion, and multivoxel MR spectroscopy sequences, were performed after every 6-week cycle of treatment or at the time of neurological deterioration.

### Statistical analysis

Statistical analyses were performed by using R statistics base package (www.r-project.org) and its libraries. Two-tailed Wilcoxon Rank Sum test with continuity correction was used to determine whether two independent groups of data were statistically different from each other. Graphical figures were constructed using R.

## Results

**Figure 1:**
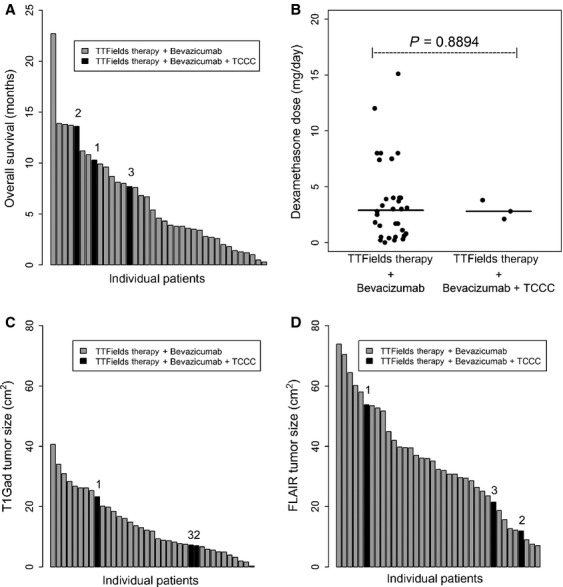
The OS of individual patients, their median daily dexamethasone usage, and their tumor size. (A) Waterfall plot of the OS of individual patients indicating that our three patients treated with NovoTTF-100A, bevacizumab, and TCCC all lived longer than the average. There was a trend suggesting that the median OS of these three patients, 10.3 (range 7.7–13.6) months, was longer than the median OS of the cohort treated with only NovoTTF-100A and bevacizumab, 4.1 (range 0.3–22.7) months (*P* = 0.0951). (B) The median daily dexamethasone dose between these two cohort did not differ, 2.8 (range 2.1–3.8) and 3.0 (0.0–15.0) mg, respectively (*P* = 0.8894). (C) Although the number in the cohort treated with NovoTTF-100A, bevacizumab, and TCCC is small in comparison with that treated with only NovoTTF-100A and bevacizumab, there does not appear to have a difference between the median tumor size in the two groups. Waterfall plot of the tumor size as measured by T1-weighted gadolinium-enhanced (T1Gad) MRI, median size 7.3 (7.0–23.3) versus 12.2 (0.3–40.6) cm^2^, respectively (*P* = 0.7809). (D) Waterfall plot of the tumor size as measured by fluid attenuated inversion recovery (FLAIR) MRI, median size 21.5 (11.9–53.8) versus 35.2 (7.0–90.9) cm^2^, respectively (*P* = 0.5043). Patients 1, 2, and 3 correspond to the MRI images displayed in Figures2, 3, and 4, respectively.

The clinical characteristics of both cohorts are listed in Table1. The cohort treated with NovoTTF-100A, bevacizumab, and TCCC (*n* = 3) did not differ significantly from the rest of the cohort treated with NovoTTF-100A and bevacizumab only (*n* = 34). The respective daily dexamethasone used in both groups were equivalent, median 2.8 (range 2.1–3.8) versus 3.0 (range 0.0–15.0) mg (*P* = 0.8894, Fig.1), as well as their respective CD3, median 774 (325–1382) versus 733 (70–1458) cells/mm^3^ (*P* = 0.4972), CD4, median 504 (254–995) versus 414 (25–788) cells/mm^3^ (*P* = 0.2861), and CD8 T lymphocyte counts, median 231 (229–343) versus 302 (44–1039) cells/mm^3^ (*P* = 0.9227). The bi-dimensional tumor size appeared to be smaller in the cohort treated with NovoTTF-100A, bevacizumab and TCCC as compared to the cohort treated with NovoTTF-100A and bevacizumab only, median 7.3 (range 7.0–23.3) cm^2^ versus 12.2 (range 0.3–40.6) cm^2^ as measured by T1Gad images and median 21.5 (range 11.9–53.8) cm^2^ versus 35.2 (range 7.0–90.9) cm^2^ as measured by FLAIR, but they were not statistically significant (Fig.1).

**Table 1:** Comparison of patient characteristics between the two cohorts (NovoTTF-100A + bevacizumab vs. NovoTTF-100A + bevacizumab + TCCC)

	NovoTTF-100A + bevacizumab	NovoTTF-100A + bevacizumab + TCCC	*P*
*N*	34	3	
Baseline characteristics			
Age (range)	57 (30−77) years	56 (51–56) years	
Gender			
Male	21	2	
Female	13	1	
Karnofsky performance status			
Median	70	70	
90	7	0	
70	15	2	
60	7	1	
50	5	0	
Tumor size, bi-dimensional			
T1Gad, median (range)	12.2 (0.3−40.6) cm^2^	7.3 (7.0–23.3) cm^2^	0.7809
FLAIR, median (range)	35.2 (7.0−90.9) cm^2^	21.5 (11.9–53.8) cm^2^	0.5043
Dexamethasone dose			
Median (range)	3.0 (0.0−15.0)	2.8 (2.1–3.8)	0.8894
Absolute T-cell subsets			
CD3, median (range)	733 (70−1458) cells/mm^3^	774 (325–1382) cells/mm^3^	0.4972
CD4, median (range)	414 (25−788) cells/mm^3^	504 (254–995) cells/mm^3^	0.2861
CD8, median (range)	302 (44−1039) cells/mm^3^	231 (229–343) cells/mm^3^	0.9227
Prior therapy			
First recurrence	6	0	
Second recurrence	9	2	
Third recurrence	9	0	
Fourth recurrence	5	1	
Fifth recurrence	5	0	
Prior bevacizumab	24	3	
Outcome			
NovoTTF-100A compliance	83.5%	66.7%	0.0670
Progression-free survival, median (range)	2.8 (0.1–20.7) months	8.1 (6.4–13.2) months	0.0585
Overall survival, median (range)	4.1 (0.3–22.7) months	10.3 (7.7–13.6) months	0.0951

TCCC, 6-thioguanine, lomustine [CCNU], capecitabine, and celecoxib.

The baseline treatment characteristics and outcome in the two cohorts are different. In the cohort treated with NovoTTF-100A and bevacizumab only, 6 (18%) were treated at first recurrence, 9 (26%) were at second, 9 (26%) were at third, 5 (15%) were at fourth, and 5 (15%) were at fifth recurrence. The Karnofsky performance status (KPS) distribution included seven (21%) at 90, fifteen (44%) at 70, seven (21%) at 60, and five (14%) at 50. In contrast, one of three (33%) patients underwent NovoTTF-100A, bevacizumab and TCCC treatment at her fourth recurrence and she had a poor KPS of 60, while the other two (67%) received treatment at their second glioblastoma recurrence and their KPS was at 70. As for treatment outcome between the two cohorts, patients treated with NovoTTF-100A, bevacizumab, and TCCC had one objective response (33%) and two stable disease (67%), while the cohort received only NovoTTF-100A and bevacizumab had stable disease as their best response. There was a difference in their compliance with the NovoTTF-100A device, with 66.7% in the former and 83.5% in the latter (*P* = 0.0670), suggesting that the former cohort may be sicker than the latter. However, despite poorer compliance, The cohort that received NovoTTF-100A, bevacizumab, and TCCC had a longer PFS than the cohort treated with NovoTTF-100A and bevacizumab only, with a median PFS 8.1 (6.4–13.2) months compared to 2.8 (0.1–20.7) months, respectively (*P* = 0.0585). There was also a trend suggesting that the former cohort had longer OS than the latter (Fig.1), with a median OS of 10.3 (7.7–13.6) months compared to a median OS of 4.1 (0.3–22.7) months (*P* = 0.0951).

**Figure 2:**
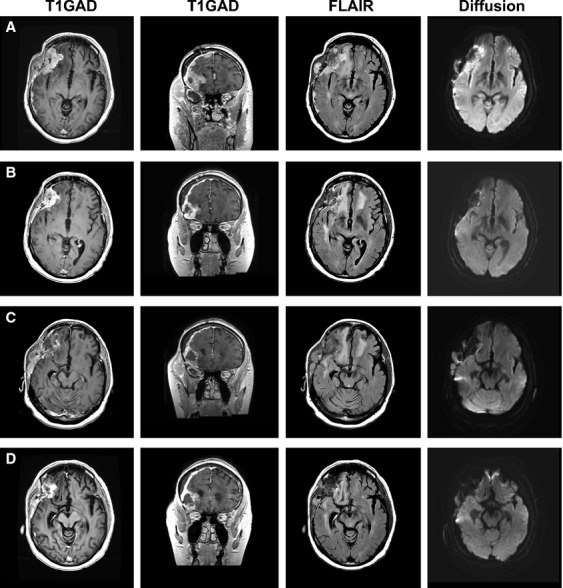
Objective response from NovoTTF-100A, bevacizumab and TCCC. (A) This panel shows baseline size of the recurrent glioblastoma in patient 1 comprising of both intracranial and extracranial components, measuring 6.4 cm × 3.0 cm (19.2 cm^2^) and 2.7 cm × 1.5 cm (4.1 cm^2^), respectively. (B) After two cycles of treatment, both intracranial and extracranial components of the tumor shrank to 4.2 cm × 2.4 cm (10.1 cm^2^) and 2.1 cm × 0.7 cm (1.5 cm^2^), respectively. There was a reduction of 50% in the bi-dimensional total tumor size, which was derived from a reduction of 47% intracranial and 63% extracranial components. (C) There was regrowth of both intracranial and extracranial components of the tumor 6 months after initiation of therapy. (D) The tumor again had a minor reduction of both intracranial and extracranial components of the tumor upon rechallenge with the addition of 6-thioguanine and lomustine for two cycles.

To better characterize the improved clinical outcome from the addition of TCCC, we performed an in-depth analysis of these individual patients. One of the patients (patient 1) treated in combination with TCCC had an objective response detected 2 months following two cycles of treatment (Fig.2). Prior to this phase of her treatment, she had failed adjuvant temozolomide, PLX3397, autologous cancer vaccine and bevacizumab. Baseline T1Gad demonstrated a large 6.4 cm × 3.0 cm (19.2 cm^2^) heterogeneously enhancing tumor in the right frontal lobe, together with dural invasion and an extracranial component measuring 2.7 cm x 1.5 cm (4.1 cm^2^). Her neurological examination was characterized by psychomotor slowing, impairment of executive function and left hemiparesis, corresponding to a KPS of 60. Her baseline lymphocyte counts were normal with 774 CD3+ (normal 578–1850), 504 CD4+ (normal 350–1100) and 231 CD8+ (normal 193–685) cells/mm^3^. Repeat head MRI after 1 month showed no detectable change in tumor size. However, 2 months after treatment initiation, her MRI demonstrated a 50% reduction in size of both intracranial and extracranial components of the tumor, to 4.2 cm × 2.4 cm (10.1 cm^2^ or 47% reduction) and 2.1 cm × 0.7 cm (1.5 cm^2^ or 63% reduction), respectively. There was also normalization of blood flow to the right frontal brain and improvement in her neurocognitive function and balance. At 2 months, 6-thioguanine and lomustine were stopped. Her dexamethasone had been decreased to 0.5 mg every other day and capecitabine and celecoxib were continued together with NovoTTF-100A and bevacizumab. Four months from initiation of treatment, her tumors returned in both intracranial and extracranial compartments accompanied by a slight increase in blood flow. She also experienced a decline in neurocognitive, executive, and motor functions. She was rechallenged with TCCC. One month after treatment, she achieved a minor response and blood flow in the right frontal brain was also normalized. Her neurological functions again improved and her immune cell counts were maintained and minimally decreased. Her tumor recurred again two months later and she exhibited further neurological decline. She died at 10.3 months after initiation of treatment and had an OS of 42.5 months from initial diagnosis.

**Figure 3:**
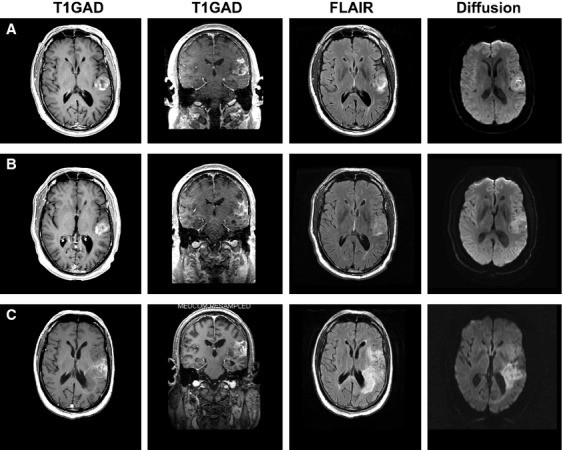
Stable disease from NovoTTF-100A, bevacizumab and TCCC. (A) At baseline, the recurrent glioblastoma in patient 2 was located in the left insula before treatment. (B) The best response was a minor reduction in gadolinium enhancement when a repeat head MRI was performed 1.5 months after initiation of treatment. (C) At the time of tumor recurrence at 13.2 months after initiation of treatment, the tumor had become diffusely infiltrative with a concomitant increase in FLAIR and diffusion signals.

The recurrent glioblastoma in patient 2, located in the left insula, had the smallest tumor size in the cohort (Fig.3). After failure from adjuvant temozolomide followed by single-agent bevacizumab, he received NovoTTF-100A, bevacizumab, and TCCC and exhibited a radiologic decrease in gadolinium enhancement 1.5 months after the first cycle. He continued treatment for 13.2 months with stable disease. He possessed a robust immune cell count profile at baseline with 1276 CD3+, 943 CD4+, and 287 CD8+ cells/mm^3^ and they were still maintained at a high level 11.0 months after treatment. He had the longest OS at 13.6 months from treatment initiation and 38.3 months from his original diagnosis.

**Figure 4:**
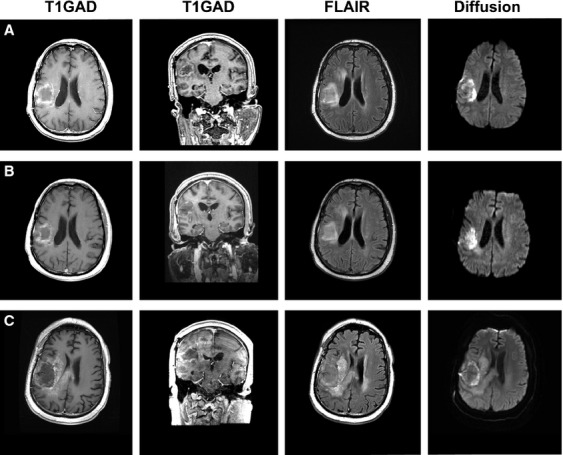
Stable disease from NovoTTF-100A, bevacizumab and TCCC. (A) At baseline, the necrotic recurrent glioblastoma in patient 3 was located in the right insula before treatment. (B) At 0.8 months after initiation of treatment, there was a slight reduction in gadolinium enhancement but no shrinkage of the tumor cyst. (C) At the time of tumor recurrence 6.4 months after initiation of treatment, his head MRI showed marked tumor invasion into the adjacent hemisphere and the corpus callosum.

The glioblastoma in Patient 3 progressed in the right insula and it manifested as left hemiparesis (Fig.4). The tumor was necrotic in appearance on MRI and he had failed adjuvant temozolomide followed by single-agent bevacizumab. The size of his tumor did not decrease during treatment with NovoTTF-100A, bevacizumab, and TCCC. His immune cell count at baseline was slightly lower than the others, with 504 CD3+, 254 CD4+, and 229 CD8+ cells/mm^3^ due to prolonged prior dexamethasone use but it was maintained at a steady level throughout his treatment. He was on treatment for 6.4 months before progression. His OS was 7.7 months from treatment initiation and 20.9 months from initial diagnosis.

## Discussion

In this study, we found that patients treated with NovoTTF-100A in combination with bevacizumab and TCCC exhibited clinical benefit compared to the cohort treated with NovoTTF-100A monotherapy in the pivotal phase III trial 6 and other patients from our institution treated with NovoTTF-100A plus bevacizumab. The control cohort treated with NovoTTF-100A and bevacizumab had a PFS comparable to the NovoTTF-100A monotherapy cohort reported in the phase III trial, median PFS 2.8 versus 2.1 months, respectively, but the OS was slightly shorter 6. However, our cohort was sicker and with poorer baseline clinical characteristics than those enrolled in the trial; 35% of our cohort had a KPS 60 or below while all of the subjects in the trial had a KPS 70 or higher. Furthermore, 56% of our cohort received NovoTTF-100A and bevacizumab at the third or greater recurrence compared to 43% of the subjects in the NovoTTF-100A monotherapy cohort in the trial. However, despite the poorer baseline patient characteristics, our patients had a comparable PFS. We have previously reported that responders treated with NovoTTF-100A monotherapy had lower daily and cumulative dexamethasone usage than nonresponders 10, suggesting that there was a potential for dexamethasone interference, and the use of bevacizumab in our patients might have helped to lower dexamethasone requirement to control neurologic deficits.

Although dexamethasone is a highly potent corticosteroid that can counteract glioblastoma-induced cerebral edema, its prolonged use can be detrimental due to severe immunosuppression 3,16. Therefore, we maintained our patients on bevacizumab to obviate dexamethasone-induced immunosuppression even though their glioblastomas progressed while on bevacizumab. Our prior safety analysis showed no overlapping toxicity when NovoTTF-100A was added to patients who were already on bevacizumab 13. Grade 3 and 4 adverse events from TCCC, which were primarily hematologic in nature, were previously shown to be manageable 14. The concurrent use of NovoTTF-100A, bevacizumab, and TCCC was well tolerated in all three of our patients.

We believe that patients receiving glioblastoma treatment need to be positioned to maximize benefit. There were a number of approaches we undertook to achieve this. First, as host immunity plays a key role in controlling cancer, including an aggressive malignancy like glioblastoma 17–19, the median daily dexamethasone dose in our three patients was minimized to 2.8 (range 2.1–3.8) mg. Importantly, we found that all the responders in the pivotal phase III trial used significantly less daily and cumulative dexamethasone than nonresponders 10. Second, among the drugs used, lomustine has the most severe myelosuppressive property and pancytopenia is often seen after multiple cycles 20. Therefore, we limited its use by giving it in a minimal and intermittent fashion, rather than continuously, in tandem cycles. Capecitabine has minimal systemic myelosuppressive side-effects because it is a nontoxic prodrug that is only metabolized by carboxylesterase and cytidine deaminase within the liver and thymidine phosphorylase in the tumor 21. Therefore, prevention of global immunosuppression in our patients was achieved by the continuation of bevacizumab to curtail dexamethasone use, together with the intermittent administration of lomustine and the selection of other drugs in TCCC that do not cause severe myelosuppression.

There was notable anti-tumor activity in our patients who were treated with NovoTTF-100A, bevacizumab and TCCC as evinced by their prolonged OS and the objective response seen in one. Patient 1 exhibited a 50% tumor reduction and experienced a 10.3 month OS after initiation of treatment, while the other two had stable disease and their OS was 13.6 and 7.7 months. There was also a trend in OS favoring these three patients when compared to the cohort treated with only NovoTTF-100A and bevacizumab, which had a median OS of 4.1 (0.3–22.7) months. Patient 1 appeared to have an intact immune function and her prior use of an experimental immune modulator PLX3397, which blocks colony-stimulating factor-1 receptor, FMS-like tyrosine kinase 3 and stem cell factor receptor 22, and/or treatment with autologous cancer vaccine may have altered the immunosuppressive stroma within her tumor, making her glioblastoma sensitized to subsequent immunomodulating therapies. Such sensitization was indicated by the dime-sized welt that appeared after injection of her autologous cancer vaccine, which she received subsequent to PLX3397 therapy, and this reaction has been correlated with prolonged PFS in patients treated with glioblastoma-derived tumor vaccines 23,24. This delayed hypersensitivity may also signify a relatively robust innate immune response in this patient that is independent of the cancer vaccine 23,24. A similar priming effect was noted in a melanoma patient with progressive systemic melanoma despite treatment with ipilimumab but had an overwhelming systemic response induced by the abscopal effect from local radiotherapy to a paraspinal mass 25.

The alternating electric fields emitted by the NovoTTF-100A device have been shown in cell culture to exert cytoplasmic stress on the endoplasmic reticulum resulting in the expression of calreticulin on the cell surface and secretion of HMGB1 9. Both have been shown to be part of an immunogenic program of cell death normally signaling viral infection that elicits cytotoxic inflammatory responses 26. Furthermore, cyclooxygenase-2 is commonly overexpressed in glioblastomas resulting in the overproduction of prostaglandin E2 and the inhibition of prostaglandin synthesis by celecoxib may therefore help to reverse tumor-induced immunosuppression 27,28. In addition, celecoxib induces endoplasmic reticulum stress by a mechanism independent of its cyclooxygenase-2 inhibitory activity 29–31 and may therefore augment the cellular stress responses induced by NovoTTF-100A.

Lastly, the efficacy of immune modulation by the alkylating agent lomustine is unknown. However, dacarbazine has been shown to upregulate the requisite NKG2D ligands on tumor cells for their subsequent elimination by natural killer cells, natural killer T cells, and CD8+ T lymphocytes 32. The addition of 6-thioguanine can potentiate the antitumor effect of lomustine 33,34. Furthermore, the antitumor effect of radiation against pancreatic xenografts was enhanced by the combination capecitabine and celecoxib, not only at the irradiated tumor but also an abscopal effect was observed on tumors outside of the irradiated field 35. This abscopal effect may be mediated by interferon-*γ* that can augment both innate and adaptive immune response against the tumor 36. Therefore, nearly all of the agents used in the NovoTTF-100A, bevacizumab, and TCCC regimen can potentially bias our responder's recurrent glioblastoma toward supporting a productive anti-tumor immune response.

There are a number of limitations in our observations. First, the number of patients treated with NovoTTF-100A, bevacizumab, and TCCC is small and we therefore cannot recommend this combination as standard clinical practice. But the findings in our patients are notable and it can serve as a basis for future clinical trials. Second, it is unclear what the relative contribution of immunosuppression in the periphery versus the tumor microenvironment has on treatment resistance in recurrent glioblastomas. Therefore, combination treatment, rather than single-agent monotherapy, will more likely effect meaningful clinical results.

In summary, our cohort with recurrent glioblastoma showed evidence of synergistic efficacy from a combination of NovoTTF-100A, bevacizumab, and TCCC. This efficacy is manifested as prolonged median PFS and OS in the cohort and an objective response observed in one patient. Future clinical trials will need to use biomarkers to investigate the relative efficacy from the individual components of the combination treatment.

## Conflict of Interest

E. T. W. and K. D. S. received an unrestricted grant from Novocure for laboratory investigation on the effect of alternating electric fields on dividing tumor cells. E. T. W. was one of the investigators in the pivotal phase III clinical trial for the NovoTTF-100A device.
